# The role of the endothelium in asthma and chronic obstructive pulmonary disease (COPD)

**DOI:** 10.1186/s12931-017-0505-1

**Published:** 2017-01-18

**Authors:** Clara E. Green, Alice M. Turner

**Affiliations:** Centre for Translational Inflammation Research, University of Birmingham, Birmingham, UK

**Keywords:** Chronic obstructive pulmonary disease, Emphysema, Chronic bronchitis, Asthma, Endothelium, Endothelial dysfunction

## Abstract

COPD and asthma are important chronic inflammatory disorders with a high associated morbidity. Much research has concentrated on the role of inflammatory cells, such as the neutrophil, in these diseases, but relatively little focus has been given to the endothelial tissue, through which inflammatory cells must transmigrate to reach the lung parenchyma and cause damage. There is evidence that there is an abnormal amount of endothelial tissue in COPD and asthma and that this tissue and its’ progenitor cells behave in a dysfunctional manner. This article reviews the evidence of the involvement of pulmonary endothelium in COPD and asthma and potential treatment options for this.

## Background

Chronic obstructive pulmonary disease (COPD) is an important smoking related condition with 10.1% prevalence (with Forced expiratory volume in one second (FEV1) < 80%) in adults over 40 years worldwide [1]. It is also an important cause of morbidity and mortality, resulting in over 3 million deaths globally in 2005 [2]. COPD is characterized by airflow obstruction which is not normally fully reversible and is generally thought to progress over time [3]. Only 20%–30% of smokers develop COPD suggesting an important role for other factors in the development of the disease [4].

Asthma is a chronic respiratory condition characterised by variable airflow obstruction and airway hyper-responsiveness (AHR) in the presence of typical symptoms such as wheeze or cough [5]. Approximately 235 million people suffer from asthma worldwide and it is the most common chronic disease in children [6].

Both asthma and COPD are disorders associated with increased inflammation [7, 8]. Therefore, much research into these conditions has concentrated on inflammatory cells, such as the neutrophil or eosinophil, but relatively little focus has been given to the endothelial tissue, through which inflammatory cells must transmigrate (transendothelial migration; TEM) to reach the lung parenchyma and cause damage. How the endothelium is functioning is therefore critical to the process of TEM and the level of inflammatory cells seen in the asthma or COPD lung. It is possible that an abnormally functioning endothelium could result in the increased inflammatory levels and tissue damage seen in asthma and COPD. This review aims to explore the evidence that the endothelium in asthma and COPD does not function normally and potential treatment options for this. By understanding the pathogenesis of obstructive lung disease further including the role of the endothelium it is possible that new treatments may be developed and the risk of asthma and COPD may be reduced.

### The endothelium

**Fig. 1:**
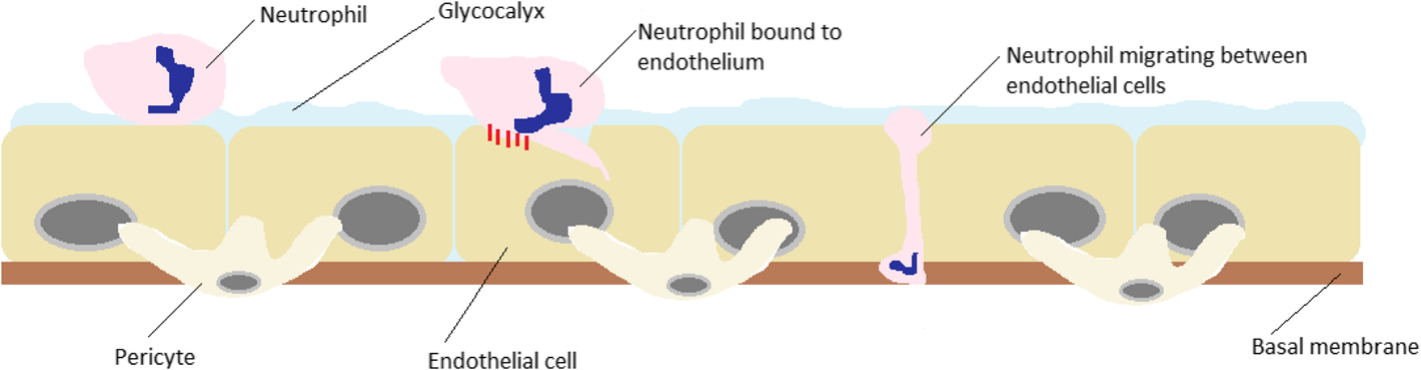
Transendothelial migration (paracellular): Neutrophil passing along the endothelium before binding to an endothelial cell via adhesion molecules (eg MAC-1). The neutrophil invaginates the endothelial cell membrane before migrating between endothelial cells

The pulmonary vasculature is critical to gas exchange in the lung, with a total pulmonary vascular surface area of 90m^2^ [9]. The entire vascular system is lined by endothelial cells which form a continuous monolayer [9]. Endothelial cells are encased by a basement membrane, a thin protein sheet (50nm thick) that consists of laminins, collagen and proteoglycans [10]. Endothelial cells are also covered on the luminal side by the glycocalyx, a network of proteoglycans and glycoproteins involved in multiple processes such as cell-cell signalling and haemostasis [11]. Finally, embedded in the basement membrane are a non-continuous layer of pericytes which are key mediators of several microvascular processes such as endothelial cell proliferation and angiogenesis [12, 13]. A diagram of the structure of the endothelium is shown in Fig. 1.

### Endothelial mechanisms of importance in asthma and COPD

#### Transendothelial migration (TEM)

Transendothelial migration (TEM) is a mechanism by which the endothelium may play a role in asthma or COPD. Neutrophils play an important role in the inflammatory response in COPD [14]. In order to reach the lung tissue neutrophils must bind to, and migrate through, the endothelium [13]. Initially neutrophils extend part of themselves (pseudopod) to invaginate the apical endothelial cell membrane. The neutrophil binds to the endothelial cell through a variety of cell surface proteins before migrating between the endothelial cells [13]. This is known as paracellular transmigration and is illustrated in Fig. 1 [13]. However, neutrophils can also transmigrate through endothelial cells in a process known as transcellular transmigration [13]. These cell surface proteins (or cell adhesion molecules) extravasate into inflamed tissue after TEM which means they are detectable in the serum [15]. Soluble cell adhesion molecule levels also correlate with cellular adhesion molecule levels thereby enabling an indirect assessment of cellular adhesion molecule levels [16].

TEM appears to be upregulated in COPD and macrophage-1 antigen (MAC-1), a protein involved in TEM is upregulated in neutrophils from COPD patients [17]. MAC-1 binds to intracellular adhesion molecule-1 (ICAM-1) on the surface of endothelial cells. Serum levels of ICAM-1 are inversely related to lung function and are also associated with increased percentages of emphysema on CT scan suggesting that this mechanism may be clinically relevant [18, 19]. Blocking the action of ICAM-1 in rodent models has also reduced pulmonary inflammation further supporting the possibility that the increase in ICAM-1 might be related to the increase in inflammation seen in COPD [20]. In addition, endothelial-leucocyte adhesion molecule-1 (ELAM-1) (another adhesion molecule involved in TEM) is also upregulated in serum in COPD patients and is particularly high in patients with chronic bronchitis further supporting the involvement of adhesion molecules in lung inflammation and COPD pathogenesis [21]. Another possible explanation for the increase in TEM in COPD is endothelial dysfunction (see below). Endothelial dysfunction is increased in COPD and appears to induce the expression of cell adhesion molecules [22].

TEM also appears to be of importance in asthma. For example, mice deficient in cell adhesion molecules L-selectin and ICAM-1 show a reduced influx of inflammatory cells into the lung and a reduction in AHR on exposure to an ovalbumin challenge [23]. In addition, in vitro studies of sensitized human bronchial tissue have demonstrated an increase in the expression of endothelial adhesion molecules (such as ICAM-1) in response to allergen exposure [24]. Gosset et al. also showed an increase in endothelial adhesion molecules in bronchial biopsies of patients with allergic asthma in comparison to controls [25]. Cell adhesion molecules in eosinophils also appear to be upregulated in asthma. Ohkawara et al. showed that eosinophils in bronchial biopsies from asthmatic patients strongly expressed MAC-1, Lymphocyte function-associated antigen 1 (LFA-1) and Very Late Antigen-4 (VLA-4) [26]. In a similar way to COPD, inflammation and underlying endothelial dysfunction could also potentially provide an underlying cause of increased adhesion molecule levels seen in asthmatic patients [20, 22].

The absolute level of adhesion molecules may not be the only factor of importance in TEM in asthma. Different alleles of adhesion molecules may predispose to asthma. For example the PECAM-1 (platelet endothelial cell adhesion molecule-1) 125 Val/leu polymorphism is more frequent in asthma patients in comparison with controls [27].

In addition to inflammation and endothelial dysfunction, another possible mechanism of increased TEM in asthma is upregulation of chemokines. Endothelial cells in asthma appear to increase production of chemokines to attract and activate circulating eosinophils. For example, Eotaxin messenger ribonucleic acid (mRNA) expression is increased in endothelial cells from bronchial biopsy specimens in asthmatic patients and levels are associated with AHR [28]. Pulmonary endothelial tissue transglutaminase 2 (TG2) is upregulated in asthma and appears to be required for eosinophil recruitment to the lungs. Mice with endothelial deficient TG2 show a reduction in lung eosinophil levels in response to an allergen challenge [29].

In summary, TEM appears to be upregulated in obstructive lung diseases and an increase in endothelial adhesion molecules is seen in both COPD and asthma. This is likely to play a role in the increased influx of inflammatory cells seen in both conditions and therefore may be important in the development of inflammation and the pathogenesis of obstructive lung disease.

### Endothelial apoptosis

Apoptosis (programmed cell death) is a highly ordered process which eliminates damaged or unwanted cells [30]. In the 1950s Liebow demonstrated that alveolar septa in COPD patients were almost avascular. This led to the hypothesis that vascular atrophy resulted in the destruction of alveoli [31]. Supporting this concept, increased levels of apoptotic endothelial cells have been identified in the lungs of patients with COPD [32]. Endothelial cells in COPD patients also demonstrate intranuclear staining of fragmented DNA in comparison to controls providing further evidence for this [33].

Animal studies provide support for apoptosis of endothelial cells resulting in emphysema. For example, it is possible to induce emphysema in rodents by deliberately causing endothelial apoptosis by blockade of vascular endothelial growth factor (VEGF) [34]. When the rodents were given a caspase (proteins involved in apoptosis) inhibitor VEGF inhibition no longer resulted in emphysema suggesting that apoptosis of endothelial cells may be key in emphysema development [34].

In addition to VEGF other mechanisms have been proposed for the development of endothelial apoptosis in COPD patients. For example, Noe et al. demonstrated that Cystic Fibrosis Transmembrane Regulator (CFTR) in human pulmonary endothelial cells was required for stress-induced apoptosis. CFTR inhibition resulted in the attenuation of endothelial apoptosis in response to treatment of cells with staurosporine or hydrogen peroxide [35]. Alpha-1-antitrypsin (A1AT) has also been shown in vitro to prevent caspase-3 activation and therefore apoptosis in pulmonary endothelial cells [36]. This would clearly be a possible relevant mechanism in emphysema development in alpha-1-antitrypsin disease (A1ATD) patients. However, smoking can induce post-translational modification of A1AT which reduces its activity suggesting that this mechanism may also be important in emphysema development in A1AT sufficient individuals [37].

Whole lung gene expression studies which have demonstrated that gene expression appears to differ between emphysematous tissue and normal lung have shown that angiogenesis-related genes are down-regulated in emphysema. Also, SERPINF1 (an antiprotease) was elevated in severe emphysema and this acts as an angiogenesis inhibitor by inducing endothelial cell apoptosis [38]. Therefore, perhaps changes in endothelial gene expression might underlie the development of emphysema. Another gene which might also be of importance is xanthine oxidase, a ROS-generating enzyme. Transcription of this gene is higher in vitro in pulmonary microvascular endothelial cells which are exposed to tobacco smoke condensate (TSC). The increased oxidative stress in response to xanthine oxidase upregulation could result in direct cell damage and apoptosis [39].

In conclusion, vascular atrophy due to endothelial apoptosis may contribute to the destruction of alveoli and consequently the development of emphysema. It may therefore be an important mechanism of COPD pathogenesis in this subgroup of patients.

### Endothelial cell senescence

Senescent cells are unable to progress through the cell cycle and divide, but remain metabolically active [40]. Senescence occurs due to telomere shortening (replicative senescence) and other, telomere-independent signals such as DNA damage or oxidative stress [41]. Replicative senescence is mediated via the p21 pathway and telomere-independent signals via the p16 pathway [42]. Lung tissue from COPD patients has an increased percentage of senescent endothelial cells and cultured pulmonary endothelial cells develop replicative senescence earlier [42]. Pulmonary endothelial cells from COPD patients have reduced telomerase activity, shorter telomeres and higher p21 and p16 levels earlier than similar cells from control subjects [42]. Oxidative stress and cigarette smoke both appear to be important in the pathogenesis of senescence in other pulmonary cells and thus are likely causes of senescence in the pulmonary endothelium [41, 43]. Senescent pulmonary endothelial cells also release more inflammatory markers and therefore are important in the increased level of inflammation seen in COPD patients [42].

### Vascular endothelial growth factor (VEGF)

A reduction in endothelium in patients with emphysema may be caused by reduced levels of Vascular Endothelial Growth Factor (VEGF) [44]. VEGF is a highly specific growth factor for endothelial cells that is produced in response to hypoxia [45]. It induces both cell proliferation and migration and prevents endothelial cell apoptosis [45]. VEGF levels might be reduced in such patients as Hypoxia Inducible Factor-1α (HIF-1α), a major transcription factor of VEGF, is also reduced in patients with emphysema. HIF-1α mediates cellular and systemic responses to hypoxia and binds to the hypoxia responsive element (HRE) on VEGF [46, 47]. Levels of HIF-1α and VEGF may be related to disease severity: both are correlated with FEV1 percentage predicted in patients with emphysema [46]. In addition, other studies have demonstrated that microRNAs -199a-5p and -34a (small non-coding RNAs that regulate gene expression) were both increased in lung tissue from COPD patients in comparison with controls. Transfection of human microvascular endothelial cells with these microRNAs resulted in decreased HIF-1α expression suggesting that epigenetic changes in COPD may also be important in COPD development [48].

Interestingly, similar studies looking at the expression of HIF-1α and VEGF in patients with chronic bronchitis (rather than emphysema) have shown HIF-1α and VEGF are increased in this patient group [49]. This suggests that the endothelium might be involved in different ways depending on the clinical presentation of COPD. Kanazawa et al. similarly found increased VEGF in sputum of patients with chronic bronchitis but decreased levels in patients with emphysema. There was a negative correlation between FEV1 and VEGF in the chronic bronchitis group but a positive association between VEGF and gas transfer (DLCO) in the emphysema group [50]. It is possible that the increased VEGF increases bronchial vascularity and leakage of plasma proteins resulting in airway narrowing in the chronic bronchitis group. However, increased VEGF might also prevent endothelial apoptosis and emphysema thus preserving gas exchange [51].

There is also evidence that vasculature may be altered in the airways of patients with COPD, in addition to their peripheral lung tissue – several groups have shown an increased vascular area in the airways of patients with COPD [52, 53]. It is possible that this might contribute to airway narrowing [53].

Patients with asthma also demonstrate increased VEGF expression in a similar way to chronic bronchitis patients. For example, VEGF mRNA levels are increased in endobronchial biopsies in patients with asthma in comparison to normal controls [54]. VEGF is also increased in induced sputum specimens in asthmatic patients and is negatively correlated with FEV1 [55]. Certain polymorphisms of VEGF (such as rs4711750 and rs3025038) also appear to confer an increased risk of asthma and are related to lung function [56, 57]. It is possible that this is due to different ratios of active and inhibitory isoforms of VEGF.

Again, in a similar way to chronic bronchitis, patients with asthma have increased vasculature in their airways which might be important in the development of airway narrowing [53]. Bronchial biopsies in asthma patients consist of more vessels than control patients [58]. The levels of vessels are related to disease severity suggesting that vascular remodelling increases as asthma severity worsens. These vessels are also associated with marked eosinophil recruitment [58]. As increased vasculature is seen in the airways of patients with mild asthma it is possible that vascular remodelling may be important in the early development of the disease [59]. Animal models suggest that the vascular remodelling may be in response to allergen exposure [60].

### Endothelial dysfunction

#### Endothelial dysfunction in COPD

In addition to altered levels of endothelium in patients with COPD, the endothelium appears to behave in a dysfunctional manner. Endothelial dysfunction is defined as disturbed endothelial dependent vasodilatation. It results in a breakdown of the microvascular endothelial barrier and loss of the anti-adhesive and anti-thrombotic functions of the endothelium [61]. Animal studies show that endothelial dysfunction appears to occur in subjects exposed to smoke before emphysema develops. This suggests that endothelial dysfunction may be important in the pathogenesis of COPD [62]. Supporting this theory Peinado et al. demonstrated that endothelial dysfunction is already present in the pulmonary arteries of patients with early COPD suggesting that this process occurs at the start of the disease process [63]. Endothelial dysfunction is associated with severity of COPD and is related to FEV1 [64–66]. Dysfunction is also related to clinical outcomes: patients with increased endothelial dysfunction have reduced 6 minute walk test (6MWT) results and a worse overall prognosis [67, 68]. Endothelial dysfunction is also increased in patients with exacerbations of COPD [69, 70]. Therefore it has been postulated that increased endothelial dysfunction may induce the development of systemic atherosclerosis and therefore the increased cardiac events seen in these patients [68].

#### Flow mediated dilation as a measurement of endothelial dysfunction

Endothelial dysfunction was previously measured by arterial catheterization to identify the response of the artery to acetylcholine. Patients with endothelial dysfunction respond with vasoconstriction rather than vasodilatation as expected [71]. However, due to the invasive nature of this technique flow mediated dilation (FMD) of the brachial artery was developed as an alternative measurement of endothelial dysfunction [72]. FMD looks at the response of the brachial artery to reactive hyperaemia using Doppler ultrasound and can be used as a surrogate measure of more central endothelial dysfunction [72]. It is reproducible both within and between days when repeated measures are made in COPD patients [73] and associated with FEV1 and percentage of emphysema on CT scan [65]. These associations were independent of smoking and other major causes of endothelial dysfunction. The relationship between FMD and FEV1 is explained by the percentage of emphysema on CT. This suggests that endothelial dysfunction might be involved in emphysema pathogenesis and COPD. [65] FMD is also able to detect changes in endothelial function in response to exacerbations: patients with exacerbations have worse endothelial function although this tends to improve after recovery from the acute episode [74].

#### Other measurements of dysfunction

In addition to FMD endothelial dysfunction can also be measured by serum markers. For example, one can look at the blood level of von Willebrand factor (vWF) as an indication of endothelial dysfunction [75]. This is a glycoprotein synthesized by endothelial cells, with increased levels being related to worsening endothelial dysfunction. Elevated vWF levels have been found in patients with COPD exacerbations implying endothelial damage occurs during these episodes [70]. Endothelial microparticles (EMPs) in blood can also be used as a measurement of endothelial dysfunction and are related to FMD [76]. EMPs are membrane vesicles which are shed by activated or apoptotic endothelial cells [69]. Gordon et al. have demonstrated that EMPs with apoptotic characteristics are increased in smokers with signs of early lung damage (normal spirometry, low DLCO) in comparison to controls [77]. This supports the hypothesis that endothelial apoptosis is an early event in the development of emphysema. EMP levels are increased in patients with COPD who have frequent exacerbations [69] and also predict patients with rapid FEV1 decline [78]. EMPs are positively correlated with the severity of emphysema in patients with COPD again suggesting that endothelial apoptosis might be an underlying mechanism of emphysema [79].

Nitric oxide (NO) is reduced in endothelial dysfunction due to a reduction in production and/or inactivation of NO synthase by ROS [75]. Maricic et al. demonstrated both increased vWF and reduced exhaled NO levels in patients with COPD [75]. Exhaled NO is also reduced in severe COPD (especially with pulmonary hypertension) in comparison to patients with mild COPD suggesting it could be useful in assessing the severity of disease [80]. Similarly Cella et al. showed plasma NO levels were also reduced in COPD as well as other markers of endothelial function such as thrombomodulin (an endothelial surface marker that binds and inactivates thrombin) [81]. However, the data for NO levels in COPD is conflictual which may limit its’ use as a tool for monitoring endothelial dysfunction. For example, during exacerbations of COPD exhaled NO appears to increase [82]. Other groups have also demonstrated a negative correlation between exhaled NO and lung function [83]. Increased plasma NO levels have also been reported in COPD [84]. Therefore, prior to any use of NO in monitoring endothelial dysfunction in the clinical setting in COPD further work must be done to clarify the exact role of NO in endothelial dysfunction and its significance.

^123^I-metaiodobenzylguanidine (^123^I-MIBG) is an analogue of guanethidine and is actively taken up and metabolized by the lungs through a sodium-dependent channel into the pulmonary endothelium. Therefore, scintigraphic analysis of ^123^I-MIBG in the lungs can be used to provide information on how well the pulmonary endothelium is functioning. The washout rate of ^123^I-MIBG is reduced in COPD patients suggesting injury to the microvascular pulmonary endothelium. Interestingly ^123^I-MIBG washout rate was also correlates with the severity of COPD (using FEV1 and DLCO) providing further support for endothelial damage underlying COPD development [85].

Finally, endothelial dysfunction of the airways can be measured specifically using airway blood flow (Qaw) measurements which should increase in response to inhaled albuterol. Patients with COPD have reduced response to albuterol suggesting endothelial dysfunction in their airways. The responsiveness to albuterol does increase after exposure of fluticasone/salmeterol for 4–6 weeks suggesting that current inhaled therapy might help improve underlying endothelial dysfunction [86].

#### Endothelial dysfunction and cardiovascular disease in COPD

It is known that the prevalence of cardiovascular disease in patients with COPD is greater than controls. The risk of cardiovascular mortality also appears to be increased in COPD patients [87]. It is not certain why this relationship exists although both diseases are related to smoking [88]. However, as FEV1 percentage predicted is independently associated with cardiovascular mortality risk it is unlikely that the relationship between COPD and cardiovascular disease is a result of smoking alone [89]. It is possible that endothelial dysfunction in COPD might provide a possible cause of increased cardiovascular disease. A recent systematic review of 22 studies has demonstrated that patients with COPD have increased levels of endothelial dysfunction in addition to increased levels of subclinical cardiovascular disease such as increased carotid intima media thickness (cIMT). The majority of the studies included in the review also accounted for smoking suggesting a link between COPD, endothelial dysfunction and subclinical cardiovascular disease that could not be explained by smoking alone [90]. A possible reason for this link is the reduced levels of soluble receptor for advanced glycation end-products (sRAGE) seen in COPD patients. sRAGE have anti-atherogenic properties and are significantly positively associated with FMD levels in COPD patients [91]. This reduction may therefore help to explain the increase in cardiovascular disease risk seen in COPD.

#### Endothelial dysfunction in asthma

The evidence for endothelial dysfunction in asthma is not as great as that in COPD. However, one study has demonstrated that asthma patients have reduced FMD levels in comparison to controls [92]. FMD was also associated with disease severity suggesting that endothelial function worsens as the disease progresses in a similar manner to COPD [92].

Qaw is increased in patients with asthma which is likely related to the increased vascularity seen in the airways in asthma [93–95]. However, in a similar way to COPD patients with asthma have a blunted Qaw response to albuterol suggesting that the endothelium in the airways is also dysfunctional [93–95].

### Endothelial progenitor cells

Endothelial progenitor cells (EPCs) act to repair endothelial injury and replace dysfunctional endothelium after being mobilized from the bone marrow to circulating blood [96]. Therefore, circulating EPCs provide a way to monitor endothelial damage. Animal studies have demonstrated that EPC levels are increased in rat models of emphysema in comparison to controls [96]. Conversely, in human studies, COPD patients appear to have reduced numbers of EPCs compared to controls [97]. When you look at COPD patients in isolation, however, patients with worse endothelial function have greater number of EPCs suggesting that vascular damage in these patients is stimulating the release of EPCs from the bone marrow [98]. It is possible that the endothelial function and EPC release may follow different pathways in COPD patients as similar studies in healthy controls show positive correlations between EPCs and FMD [99].

There is also evidence that EPCs do not function normally in patients with COPD. EPCs isolated from COPD patients had reduced proliferation rates and formed fewer clusters in vitro compared to control patients. EPCs from COPD patients had reduced chemotaxis levels and were less able to form tubular structures (in Matrigel angiogenesis studies) than control EPCs suggesting that their ability to repair endothelium was reduced. Expression of platelet/endothelial cell adhesion molecule-1 (PECAM-1), an adhesion molecule necessary for endothelial migration and junctional integrity on the surface of EPCs was also reduced in COPD patients further suggesting their dysfunctional nature. This was supported in animal studies by the same group who showed that fewer EPCs from COPD patients attached to injured arterial intima in mice compared to controls [100]. Human studies also support the dysfunctional nature of EPCs in COPD. One study looked at the levels of EPCs in patients with and without COPD before and after lung resection surgery. In control patients EPCs increased after surgery suggesting a normal response to injury. However, in COPD patients there was not an increase in EPCs suggesting the mobilization capacity of EPCs in COPD patients is reduced [101]. One explanation for the dysfunctional capacity of EPCs in COPD patients is the increased level of beta-2 adrenergic receptors (β2ARs) on EPCs from COPD patients. The increased β2AR level appears to alter the migration and proliferation seen in these cells [102].

In contrast to COPD EPCs appear to be upregulated in patients with asthma [103]. EPCs derived from patients with asthma also appear to have a higher proliferative capacity and an increased ability to form tubular structures in vitro in comparison to controls [103]. Recruitment of EPCs appears to be related to allergen challenge: in murine models of asthma circulating EPC levels were increased in response to an allergen challenge and mobilised to the lungs [103]. Vessel density in the lungs was also increased within 48 h of the challenge suggesting that EPC recruitment is important for the increased vascularity seen in asthma [103]. Similar findings have been seen in humans. On exposure to inhaled allergens, asthma patients show increased EPC mobilisation from the bone marrow [104]. Another study also demonstrated increased EPC levels in sputum 24 h after an allergen challenge. There was also an associated increase in the number and diameter of blood vessels in lung biopsy specimens further supporting the hypothesis than EPC mobilisation is important to the development of increased vascularity in lung tissue in asthma [105].

### Small vessel disease

Smoking is known to have widespread effects on the microcirculation and may result in microvascular disease in various organs such as the eye, heart and kidney [106]. It has therefore been postulated that a similar process might occur in the lungs in COPD. In support of this, studies using magnetic resonance imaging (MRI) have demonstrated that pulmonary microvascular blood flow appears to be reduced in COPD [107]. These changes were apparent in patients with mild COPD and were worse in patients with severe COPD. This suggests that microvascular disease may represent an early part of the development of COPD and potentially is important in driving the progression of COPD to more severe disease. There is evidence that pulmonary perfusion is associated with the number of small pulmonary vessels present [108] and, in a similar fashion to perfusion, the number of small vessels appear to be reduced in the COPD lung. Studies have shown that the percentage of vessels less than 5mm (%CSA <5) on CT scans appears to be reduced in patients with emphysema and is related to disease severity [109, 110]. In a similar way to microvascular blood flow, reduction in %CSA < 5 also occurs in patients with mild disease suggesting that this may have a role in the pathogenesis of emphysema [110]. %CSA < 5 also appears to be associated with exacerbations of COPD: patients with a history of exacerbations have a significantly lower %CSA < 5 in comparison to patients without exacerbations [111]. Perhaps exacerbations result in tissue and vessel damage resulting in reduced %CSA < 5 although it is possible that a low %CSA < 5 is a risk factor for the development of exacerbations [111]. The underlying mechanism for this is not known and would require further study. The potential mechanisms for the development of small vessel disease in COPD have been investigated recently in vitro. One team looked at the expression of Krüppel-like factor 5 (KLF5) in the small pulmonary vessels in COPD patients. KLF5 is a zinc-finger transcription factor which plays a role in the vascular remodelling seen in cardiovascular diseases. In a similar way to cardiovascular disease KLF5 expression was increased in COPD pulmonary vessels suggesting a possible role in the small vessel disease seen in these patients [112].

### Angiopoietins

Angiopoietins and their receptors (Tie-1 and Tie-2) are involved in the late phases of angiogenesis. Angiopoietin 1 (Ang-1) is proangiogenic and acts in the development of vascular networks [113]. Ang-2 appears to be an antagonist of Ang-1 and Tie-2, but can also act to enhance the proliferation and migration of endothelial cells [114, 115]. Studies have shown that the levels of angiopoietins appear to be altered in patients with COPD. For example, García-Lucio et al. showed that the expression of Ang-2 in pulmonary arteries appeared to be increased in COPD patients in comparison to healthy smokers [116]. Similarly, Bessa et al. showed increased Ang-2 levels in patients with COPD in induced sputum [117]. Ang-2 was also associated with vascular permeability in COPD patients suggesting that the increase in Ang-2 might stimulate leakage from vessels in COPD [117]. Interestingly, the levels of Ang-2 in the blood appear to be higher in patients with moderate COPD rather than severe COPD [113]. This could possibly suggest that an increase in Ang-2 might be important in the early stages of vascular remodelling in COPD, but not in later phases of the disease when changes in vasculature are already established. However, it is possible that levels of Ang-2 fluctuate in patients with COPD during exacerbations. Nikolakopoulou et al. demonstrated that serum Ang-2 levels are increased at the onset of COPD exacerbations and correlated with C-reactive protein (CRP) levels. The levels of Ang-2 decreased after a week of treatment. Patients with poorer outcomes also had significantly higher Ang-2 levels [118]. This suggests that Ang-2 might be a useful biomarker for COPD exacerbations and might help clinicians identify patients at risk of worse outcomes at the start of an exacerbation. Supporting this finding, another group found that blood levels of Ang-1 are reduced during COPD exacerbations and increased when patients are clinically stable [119]. As Ang-2 acts as an Ang-1 antagonist perhaps increased Ang-2 levels in these patients resulted in a reduction of Ang-1.

Angiopoietins also appear to play a role in asthma. Levels of both Ang-1 and Ang-2 in sputum are increased in stable asthmatic patients in comparison to controls. Smoking also increased angiopoietin levels in the asthma group [120]. Angiopoietins appear to be related to disease severity in asthma. Sputum levels of both Ang-1 and Ang-2 are significantly increased in severe refractory asthma patients in comparison to patients with moderate asthma [121]. This might suggest that they are important in the vascular remodelling seen in asthmatic patients. Similarly, serum Ang-1 and Ang-2 levels are also increased in asthma patients in comparison to healthy controls. However, only serum Ang-2 appears to be related to disease severity and is higher in refractory asthma cases. Serum Ang-2 (but not Ang-1) also correlates with parameters of severe asthma including number of exacerbations, emergency medical trips and number of hospitalizations [122]. Serum Ang-2 is positively correlated with exercise-induced bronchoconstriction whereas Ang-1 does not show this association. This suggests that Ang-2 might have a more important role in remodelling and disease pathogenesis seen in asthma and could potentially have use as a biomarker for severe asthma [123]. In a similar way to COPD, angiopoietins also appear to be altered acutely during asthma exacerbations. Lee et al. demonstrated that plasma Ang-2 levels were increased during exacerbations in comparison to patients with stable asthma whereas Ang-1 levels were lower during exacerbations. Ang-2 levels also correlated with the level of eosinophils and neutrophils, two important inflammatory cells involved in asthma [124].

### Summary

**Fig. 2:**
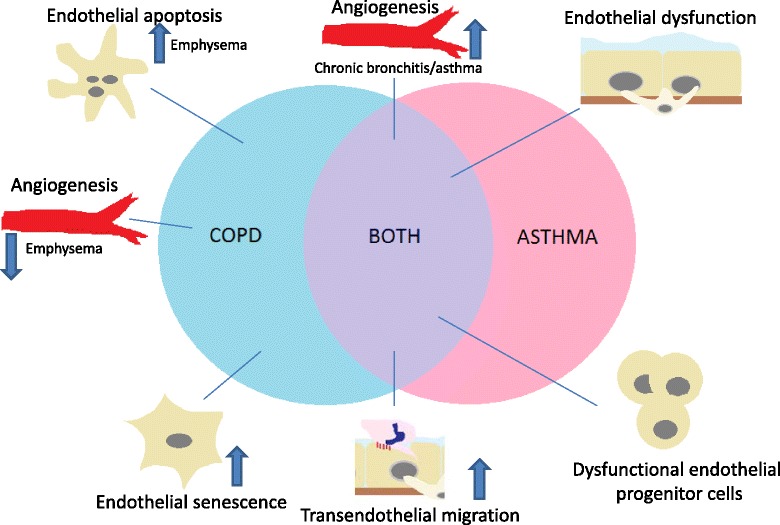
Different endothelial mechanisms important in COPD and asthma pathogenesis

In conclusion, the endothelium appears to behave in an abnormal fashion in COPD and asthma. Multiple pathways involving the endothelium may have importance in both conditions. In some cases such as TEM, pathways appear to behave in a similar fashion in COPD and asthma. However, other pathways behave differently depending on the phenotype of the patient. For example, increased angiogenesis in asthma/chronic bronchitis and decreased angiogenesis in emphysema. A summary of the mechanisms involved in endothelial dysfunction in COPD and asthma can be seen in Fig. 2.

### Pathogenesis of endothelial dysfunction

#### COPD

Endothelial dysfunction may be a result of the increased levels of oxidative stress seen in COPD. Patients with COPD and low levels of FMD show improvements in FMD when given anti-oxidants [125]. The RhoA/Rho-kinase pathway which is upregulated in patients with COPD may also result in endothelial dysfunction. RhoA is a small G-protein and Rho-kinase is its’ downstream effector. This pathway is important in a variety of cell functions including migration and proliferation. Levels of RhoA and Rho-Kinase are associated with the level of endothelial dysfunction in patients with COPD [126]. Angiotensin-converting enzyme (ACE), a regulatory protein with both vascular and collagenolitic effects has different variants. The D variant is associated with both endothelial dysfunction and number of exacerbations in patients with COPD and thus may also play a role in the development of endothelial dysfunction [127].

As systemic inflammation is increased in COPD patients studies have been performed to investigate whether the level of inflammation seen in these patients is related to endothelial dysfunction. Eickhoff et al. demonstrated that FMD was associated with C-reactive protein and leukocyte levels suggesting an underlying association between inflammation and endothelial dysfunction [64]. However, other studies have had conflicting results and have not been able to demonstrate a relationship between endothelial dysfunction and inflammation [128]. It is possible that this might be a result of the patients included in the different studies. Eickhoff et al. included COPD patients without comorbidities whereas other studies included patients with comorbidities such as cardiovascular disease which may have masked the effect of COPD and inflammation.

Insulin resistance may also have a role in the development of endothelial dysfunction in patients with COPD. One study followed up COPD patients over the course of a year. During this time FMD decreased and fasting blood glucose increased. FMD was significantly negatively correlated with fasting blood glucose suggesting that patients with worse glucose control also had worse endothelial function [129]. Interestingly, other studies have demonstrated that uncontrolled diabetes mellitus is also associated with worse lung function in COPD patients. This suggests that insulin resistance might have several roles in COPD pathogenesis [130].

### Asthma

There is a lack of evidence for why patients with asthma might develop endothelial dysfunction. However, one study demonstrated that vascularity in bronchial biopsy specimens was reduced in asthma patients after a 6 month course of inhaled corticosteroids (ICS) [131]. This suggests that inflammation may be an important cause of the increased vascularity and vascular remodelling seen in these patients. These findings were repeated in another study which also demonstrated that VEGF levels reduced after a 6 month ICS trial thereby suggesting that inflammation in asthma might result in increased airway vascularity through upregulation of VEGF [132].

### Potential for endothelial based treatments

#### COPD

Many of the above pathways have potential treatments associated with them. For example, as above, patients with COPD and reduced FMD have shown improvement in FMD with the use of anti-oxidants [125]. Ginkgo biloba extract (EGb) an agent with anti-oxidant properties has also been shown to reduced cigarette smoke extract (CSE) induced apoptosis in pulmonary endothelial cells by upregulation of haem oxygenase-1 (HO-1, a stress-responsive protein) [133]. Patients on long-term ICS have both lower levels of VEGF in bronchial tissue [134] and reduced endothelial dysfunction compared to patients not on ICS [135]. Therefore, targeting inflammation and may be important to improve endothelial dysfunction in these patients [135]. A randomised control trial (RCT) of statin therapy in COPD patients also demonstrated that statins reduced the level of systemic inflammatory markers seen in these patients and also improved endothelial function in patients with evidence of raised systemic inflammation [136]. The ACE D variant is associated with increased production of ACE suggesting that perhaps ACE inhibitor drugs (already available for other conditions such as hypertension) may provide another treatment option for this subgroup of patients [127]. Rho-kinase inhibitors have also improved NO release from endothelial cells in vitro suggesting that blocking this pathway may provide another means to improve endothelial dysfunction [137]. As FMD is associated with poor glucose control screening COPD patients for fasting blood glucose and controlling abnormal glucose levels might provide another method of improving endothelial function in these patients [129].

It is possible that some of the treatments already available for COPD might be able to improve endothelial functioning. For example, one study observing endothelial function in COPD patients demonstrated that patients with improved 6MWT scores had improved FMD levels [128]. Therefore, it is possible that pulmonary rehabilitation courses could provide one way of improving endothelial function. In vitro studies have also demonstrated treating the increased level of β2AR on EPCs with β2 antagonists improves the proliferation and migratory capacity of these cells [102]. One RCT looking at endothelial function in COPD patients observed an improvement in FMD after lung volume reduction surgery (LVRS) (surgery to remove abnormal emphysematous tissue). It is not clear why LVRS improves endothelial functioning but one possibility is that improved cardiac function seen after LVRS might stimulate the endothelium, thus improving FMD [138].

Other studies have focused on treating the increased levels of endothelial apoptosis seen in patients with emphysema. For example, one study demonstrated that CSE induced apoptosis and decreased prostacyclin synthase levels in human umbilical vein endothelial cells (HUVECs) in vitro [139]. Prostacyclin is a known vasodilator and appears to possess anti-apoptotic effects. Apoptosis on exposure to CSE was prevented by treatment with the prostacyclin analogue beraprost sodium [139]. Similarly, another treatment known to upregulate prostacyclin levels, Honokiol (a compound extracted from a Chinese medicinal herb) also reduced apoptosis in endothelial cells in response to low-density-lipoprotein (LDL) in vitro [140]. Human studies have also shown lower prostacyclin expression levels in emphysematous lung compared to normal lung and demonstrated that another prostacyclin analogue (iloprost) also prevented endothelial apoptosis in vitro [141]. A summary of potential endothelial based treatments in COPD can be seen in Table 1.

**Table 1:** Potential endothelial based treatments in COPD

Pathway targeted	Drug	Outcome	Stage of testing	Reference
Anti-oxidant	vitamin C, vitamin E, α-lipoic acid	Improved FMD	Phase II	[125]
	Ginkgo biloba extract	Reduced endothelial apoptosis	In vitro	[133]
Inflammation	ICS	Reduced bronchial VEGF, reduced FMD	Phase II	[134]
	Statin	Reduced FMD	Phase II (NCT00929734)	[136]
Rho-kinase	Hydroxyfasudil	Increased NO release from endothelial cells	In vitro	[137]
Increased level of β2AR on EPCs	β2 antagonists	Improved proliferation and migration of EPCs	In vitro	[102]
Removal of abnormal tissue	LVRS	Reduced FMD	Phase II (NCT01020344)	[138]
Prostacylin	Beraprost sodium	Reduced endothelial apoptosis	In vitro	[139]
	Iloprost	Reduced endothelial apoptosis	In vitro	[141]

Where possible clinicaltrials.gov identifiers are in brackets

### Asthma

As previously mentioned, targeting the inflammation seen in asthma using ICS can reduce VEGF expression and airway vascularity [131, 132]. Animal studies also support targeting VEGF as a potential treatment strategy in asthma. For example, two inhibitors of VEGF receptor were given to a murine asthma model. Mice who received the inhibitors demonstrated reduced VEGF airway levels, reduced airway inflammation and reduced AHR [142]. This suggests that targeting VEGF might be useful for both treating underlying vascular remodelling and symptoms in asthma. Another animal study also demonstrates that reducing angiogenesis in asthma might be effective. Vascular endothelial (VE)-cadherin antibodies were given to a mouse model of asthma. VE-cadherin is an endothelial adhesion molecule important in angiogenesis. Delivery of VE-cadherin antibodies reduced angiogenesis in the mouse model, but also reduced IgE production and eosinophil airway infiltration both hallmarks of inflammation seen in asthma [143]. AHR was also reduced in response to the antibody [143].

Another potential treatment option is to target EPCs in asthma. When a chemokine receptor antagonist (AMD3100) was given to mice sensitized to ovalbumin airway pulmonary EPCs, eosinophil accumulation, vascularity and AHR was reduced [144]. However, when AMD3100 was given to mice with established lung disease although the drug reduced EPCs, eosinophil levels and vascularity, AHR was only partially reversed [144]. This shows that it is likely to be important to reduce EPC accumulation early in asthma to prevent established airway obstruction.

Finally, reducing TEM may be another treatment option for patients with asthma. Theophylline (a methylxanthine drug with bronchodilator and anti-inflammatory properties) was added to HUVECs in vitro. This reduced adhesion of eosinophils to the HUVECs and also reduced the expression of endothelial adhesion molecules such as ICAM-1 [145]. This suggests that theophylline (which is already in use in asthma) may have beneficial effects by reduced TEM of eosinophils and consequently inflammation in asthma. A similar in vitro study was also performed by exposing HUVECs to another drug used in asthma: montelukast (a cysteinyl LT1-receptor anatagonist). Montelukast also reduced eosinophil transmigration across HUVECs and may therefore partially act by reducing TEM of eosinophils in patients [146]. There is also evidence that new drugs may be of use by targeting TEM. VUF-K-8788 (a histamine H1 antagonist) reduced eosinophil adherence to HUVEC in vitro. The same drug also reduced pulmonary eosinophil accumulation and inflammation (such as perivascular oedema) in a guinea pig model of asthma [147]. A summary of potential endothelial based treatments in COPD can be seen in Table 2.

**Table 2:** Potential endothelial based treatments in asthma

Pathway targeted	Drug	Outcome	Stage of testing	Reference
Inflammation	ICS	Reduced airway VEGF and airway vascularity	Phase II	[132]
VEGF	SU5614	Reduced airway VEGF, inflammation and AHR	Murine model	[142]
	SU1498	Reduced airway VEGF, inflammation and AHR	Murine model	[142]
Angiogenesis	VE-cadherin antibody	Reduced angiogenesis, IgE production, eosinophil infiltration and AHR	Murine model	[143]
Chemokine signalling	AMD3100 (chemokine receptor anatagonist)	Reduced airway pulmonary EPCs, eosinophil accumulation, vascularity and AHR	Murine model	[144]
TEM	Theophylline	Reduced adhesion of eosinophils to endothelium	in vitro	[145]
	Montelukast	Reduced eosinophil transmigration across endothelium	in vitro	[146]
	VUF-K-8788 (Histamine H1 antagonist)	Reduced adherence of eosinophils to endothelium in vitro. Reduction of pulmonary eosinophil accumulation.	Guinea pig model	[147]

## Conclusions

Pulmonary endothelium in asthma and COPD patients appears to be altered in comparison to control subjects. There is evidence that in some COPD patients with the subtype of emphysema apoptosis of the endothelium may result in alveolar destruction and reduced gas transfer. In patients with asthma or chronic bronchitis increased VEGF and vascular remodelling in the airways may have a more important role. The endothelium also behaves in a dysfunctional manner in COPD and endothelial progenitor cells appear to be less effective at repairing the damaged and dysfunctional endothelial tissue. There is some evidence that endothelial dysfunction also occurs in asthma and endothelial progenitor cells are upregulated in these patients.

Multiple mechanisms such as inflammation may explain the underlying alteration in the endothelium in these patients and some already existing treatments could target these mechanisms and improve underlying endothelial function. Very few studies looking into treatment of endothelial dysfunction in COPD and asthma exist and more work is required to evaluate whether or not mechanisms of endothelial dysfunction researched in vitro will lead to promising treatment strategies in COPD and asthma.

## Abbreviations

%CSA <5: Percentage of vessels less than 5mm on CT scans
I-MIBG: ^123^I-metaiodobenzylguanidine
6MWT: 6 minute walk test
A1AT: Alpha-1-antitrypsin
A1ATD: Alpha-1-antitrypsin disease
ACE: Angiotensin-converting enzyme
AHR: Airway Hyper-Responsiveness
Ang-1: Angiopoietin 1
Ang-2: Angiopoietin 2
CFTR: Cystic Fibrosis Transmembrane Regulator
cIMT: carotid intima media thickness
COPD: Chronic Obstructive Pulmonary Disease
CRP: C-reactive protein
CSE: Cigarette smoke extract
DLCO: Gas transfer
EGb: Ginkgo biloba extract
ELAM-1: Endothelial-Leucocyte Adhesion Molecule-1
EMPs: Endothelial microparticles
EPCs: Endothelial progenitor cells
FEV1: Forced Expiratory Volume in one second
FMD: Flow mediated dilation
HIF-1α: Hypoxia Inducible Factor-1α
HO-1: Haem oxygenase-1
HRE: Hypoxia responsive element
HUVECs: Human umbilical vein endothelial cells
ICAM-1: Intracellular Adhesion Molecule-1
ICS: Inhaled corticosteroids
KLF5: Krüppel-like factor 5
LDL: Low-density-lipoprotein
LFA-1: Lymphocyte function-associated antigen 1
LVRS: Lung volume reduction surgery
MAC-1: Macrophage-1 Antigen
MRI: Magnetic resonance imaging
mRNA: messenger ribonucleic acid
NO: Nitrogen oxide
PECAM: Platelet Endothelial Cell Adhesion Molecule-1
PECAM-1: Platelet/endothelial cell adhesion molecule-1
RCT: Randomised control trial
SERPINF1: Serpin Family F Member 1
sRAGE: soluble receptor for advanced glycation end-products
TEM: Transendothelial Migration
TG2: Tissue transglutaminase 2
TSC: Tobacco smoke condensate
VE-cadherin: Vascular endothelial cadherin
VEGF: Vascular endothelial growth factor
VLA-4: Very Late Antigen-4
vWF: von Willebrand factor
β2ARs: beta-2 adrenergic receptors
